# Investigating the impact of alpha/beta and LET_d_ on relative biological effectiveness in scanned proton beams: An *in vitro* study based on human cell lines

**DOI:** 10.1002/mp.14212

**Published:** 2020-05-15

**Authors:** Elisabeth Mara, Monika Clausen, Suphalak Khachonkham, Simon Deycmar, Clara Pessy, Wolfgang Dörr, Peter Kuess, Dietmar Georg, Sylvia Gruber

**Affiliations:** ^1^ Department of Radiation Oncology/Christian Doppler Laboratory for Medical Radiation Research for Radiation Oncology Medical University of Vienna Vienna Austria; ^2^ University of Applied Science Wiener Neustadt Austria; ^3^ Division of Radiation Therapy Department of Diagnostic and Therapeutic Radiology Faculty of Medicine Ramathibodi Hospital Mahidol University Bangkok Thailand; ^4^ Laboratory of Applied Radiobiology Department of Radiation Oncology University Hospital Zürich Zürich Switzerland; ^5^ EBG MedAustron GmbH Wiener Neustadt Austria

**Keywords:** α/ß‐ratio, linear energy transfer, pencil beam scanning, proton therapy, RBE modeling, relative biological effectiveness

## Abstract

**Purpose:**

A relative biological effectiveness (RBE) of 1.1 is commonly used in clinical proton therapy, irrespective of tissue type and depth. This *in vitro* study was conducted to quantify the RBE of scanned protons as a function of the dose‐averaged linear energy transfer (LET_d_) and the sensitivity factor (α/ß)_X_. Additionally, three phenomenological models (McNamara, Rørvik, and Jones) and one mechanistic model (repair‐misrepair‐fixation, RMF) were applied to the experimentally derived data.

**Methods:**

Four human cell lines (FaDu, HaCat, Du145, SKMel) with differential (α/ß)_X_ ratios were irradiated in a custom‐designed irradiation setup with doses between 0 and 6 Gy at proximal, central, and distal positions of a 80 mm spread‐out Bragg peak (SOBP) centered at 80 mm (setup A: proton energies 66.5–135.6 MeV) and 155 mm (setup B: proton energies 127.2–185.9 MeV) depth, respectively. LET_d_ values at the respective cell positions were derived from Monte Carlo simulations performed with the treatment planning system (TPS, RayStation). Dosimetric measurements were conducted to verify dose homogeneity and dose delivery accuracy. RBE values were derived for doses that resulted in 90 % (RBE_90_) and 10 % (RBE_10_) of cell survival, and survival after a 0.5 Gy dose (RBE_0.5Gy_), 2 Gy dose (RBE_2Gy_), and 6 Gy dose (RBE_6Gy_).

**Results:**

LET_d_ values at sample positions were 1.9, 2.1, 2.5, 2.8, 4.1, and 4.5 keV/µm. For the cell lines with high (α/ß)_X_ ratios (FaDu, HaCat), the LET_d_ did not impact on the RBE. For low (α/ß)_X_ cell lines (Du145, SKMel), LQ‐derived survival curves indicated a clear correlation of LET_d_ and RBE. RBE_90_ values up to 2.9 and RBE_10_ values between 1.4 and 1.8 were obtained. Model‐derived RBE predictions slightly overestimated the RBE for the high (α/ß)_X_ cell lines, although all models except the Jones model provided RBE values within the experimental uncertainty. For low (α/ß)_X_ cell lines, no agreement was found between experiments and model predictions, that is, all models underestimated the measured RBE.

**Conclusions:**

The sensitivity parameter (α/ß)_X_ was observed to be a major influencing factor for the RBE of protons and its sensitivity toward LET_d_ changes. RBE prediction models are applicable for high (α/ß)_X_ cell lines but do not estimate RBE values with sufficient accuracy in low (α/ß)_X_ cell lines.

## 1 INTRODUCTION

Proton therapy (PT) is an emerging treatment modality within radiation oncology with 77 facilities currently in operation (www.ptcog.ch). Compared to most advanced treatment techniques with high‐energy photon beams, the advantageous physical ballistics of protons enable highly conformal treatments with reduced organs‐at‐risk doses, especially in the low and medium dose range, and a substantially reduced integral dose.^1^ Due to the effective sparing of normal tissues without compromised target coverage, PT is primarily applied in pediatric oncology and for malignancies close to critical anatomical structures, that is, for head‐and‐neck and skull base region as well as re‐irradiations.^2^, ^3^, ^4^

In addition to the favorable inverted depth dose profile, protons are biologically more effective than high‐energy photon beams. Hence, the relative biological effectiveness (RBE) was introduced as a conversion factor, defined as the ratio of physical dose required to yield the same biological effect.^5^, ^6^ Today's clinical practice in PT is still based on a constant RBE of 1.1, a value which was chosen conservatively in the 1960s and 1970s.^7^, ^8^ However, the knowledge gained from several studies suggests a variable, rather than a constant RBE. Fractional doses, physical beam characteristics, biological parameters, and the investigated endpoints were identified as influencing factors. Especially the increased linear energy transfer (LET) at the distal end of the spread‐out Bragg peak (SOBP) is associated with increased biological efficacy.^9^, ^10^ However, none of these parameters have so far been implemented in clinical routine, largely due to uncertainties within these complex interactions and the broad range of reported RBE values from 0.8 to 2.^11^, ^12^ These variations in RBE can be partly attributed to the use of different reference irradiation qualities, beam delivery techniques and energies, endpoint assessment at different positions within the beam line, and chosen radiobiological model systems.^11^, ^13^, ^14^, ^15^

To account for a potential variable RBE, several mathematical models have been developed to predict the RBE. All phenomenological models are empirical data‐based, generally taking into account the dose, dose‐averaged linear energy transfer (LET_d_), and tissue‐specific parameters generated from a collection of *in vitro* data with clonogenic death as biological endpoint. In contrast, mechanistic models are derived from predicting the interaction probability of particles within the biological system, including, to a varying extent, DNA double‐strand break (DSB) repair dynamics. Again, clinical implementation so far was hindered due to uncertainties in the fitting data.^16^ Concerning the beam delivery, pencil beam scanning with active or passive depth modulation has eclipsed the traditional passive scattering techniques. The latter was the basic mode of beam delivery in which almost all experimentally available RBE data were acquired. As very recently pointed out, the physical characterization of particle beams and the standardization of dosimetric reporting are essential steps to reduce uncertainties in RBE determination.^13^, ^17^ In summary, during the last decades the scientific knowledge, methodological approaches, and the mode of proton beam delivery have changed and progressed considerably.

The aim of the present study was to investigate the correlation of RBE, LET_d_, and the tissue‐specific fractionation sensitivity factor of photons (α/β)_X_ for scanned proton beams with an active energy variation in experimental conditions that mimic typical clinical scenarios (e.g., dose, energy range). More specifically, the LET_d_ dependency of the proton RBE was assessed for four human cell lines with high (≥10) and low (≤5) (α/β)_X_ values using typical clinical proton energy ranges, provided by the synchrotron at the MedAustron facility.

Our experimentally derived RBE values were compared to three phenomenological models (McNamara,^18^ Rørvik,^19^ and Jones^20^) as well as one mechanistic model (repair‐misrepair‐fixation (RMF) model^21^). These models were specially chosen according to their data sets. The McNamara (MCN) model is based on experimental data reviewed and summarized by Paganetti et al.^22^ In more detail, 285 data points were extracted from experiments with several different cell lines and LET_d_ values ranging of up to 20 keV/µm. Thus this model covers a large range of (α/β)_X_‐ as well as LET_d_ values, although the database is dominated by Chinese hamster cell lines with a low (α/β)_X_ ratio and LET_d_ values lower than 5 keV/µm. Furthermore, MCN assumes that the RBEs at extreme high and low dose levels, RBE_min_ and RBE_max_, depends on (α/β)_X_ and LET_d_. The Rørvik (RØR) model database contains 85 data points extracted from experiments with low and high (α/β)_X_ cell lines, but is, similar to the MCN model, dominated by low (α/β)_X_ cell lines. RØR has the most uniform range of LET_d_, including the highest values, from all phenomenological models tested. The RØR model assumes an (α/β)_X_‐ and LET_d_ dependency of the RBE_max_ but utilizes a constant RBE_min_ of 1. The Jones (JON) model compromises a simpler LET efficiency approach and is based on data from protons and ions, for example, helium, carbon, and neon. Different to the MCN and the RØR model, the JON model exclusively contains data points with LET_d_ larger than 5 keV/µm. In contrast to the other two phenomenological models, JON’s tissue dependency is based on the absolute values of α_x_ and β_x_ independent of each other and the input data are solely from tissues with low (α/β)_X_ values.^23^

The mechanistic RMF model, which itself is based on the double‐strand break (DSB) model,^24^ was chosen due to the availability of the DSB model in the treatment planning system (TPS, RayStation 5.99) being used in this study. The RMF model RBE estimates correspond to the exact position of the cell layers within the planned target volume (PTV).

## 2 MATERIALS AND METHODS

### 2.A. Cell cultures and procedures

Four human cell lines were chosen, which represent two *in vitro* models with a high (α/β)_X_ ratio, head‐and‐neck squamous cell carcinoma (FaDu), and normal skin keratinocytes (HaCat), as well as two models with a low (α/β)_X_, melanoma (SKMel) and prostate carcinoma (Du145).

HaCat were cultured in Dulbecco's modified Eagle medium (DMEM), supplemented with 10 % fetal calf serum (FCS), 25 mM HEPES, 1% sodium pyruvate, and 100 U/ml penicillin and streptomycin. FaDu was maintained in Roswell Park Memorial Institute (RPMI) 1640, supplemented with 10% FCS, 25 mM HEPES, and 100 U/ml penicillin and streptomycin. Du145 and SKMel were cultivated in Minimum Essential Medium Eagle (MEM), supplemented with 10 % FCS, 25 mM HEPES, 2 mM L‐Glutamine, and 100 U/ml penicillin and streptomycin.

All cells were cultured at 37°C in a humidified atmosphere with 95% air and 5% CO_2_. Cells were seeded in chamber slide flasks (Nunc™ Lab‐Tek™ II Chamber Slide™ System) with plastic slides at 2.5 to 5 × 10^5^ cells per flask 24–48 hr before irradiation to achieve 70–80% confluency at the time of irradiation. Immediately prior to the irradiation, the chamber slide flasks were filled air‐bubble free with the respective unsupplemented medium.

### 2.B. Photon and proton irradiation and dosimetry

For both, reference x‐ray as well as proton irradiation, dedicated polymethyl methacrylate (PMMA) irradiation setup were developed to accommodate for the horizontal beam geometry and to ensure standardized sample positioning (see Figures S1 and S2). Detailed dosimetric verification of the PMMA irradiation setup preceded the experiments.^25^ Cells were irradiated with 0.5, 1, 2, 4, and 6 Gy physical dose. For each experiment an equally processed, nonirradiated negative control was carried out. The negative control was filled with unsupplemented medium and left inside of the irradiation rooms (x‐rays as well as proton irradiation room) for the duration of a 6 Gy irradiation. The flask holder itself was designed so that the transition zones between PMMA and cell medium were limited (see Figure S1). This is of essential importance as dose levels can change significantly in such transition zones. The dosimetric impact caused by different cell media and water was investigated in a preceding experiment, where no significant differences were found. For each dose level and beam quality, at least three independent irradiation sessions were conducted.

#### 2.B.1. Reference x‐ray irradiation

Reference irradiation was performed in a 200 kV beam, generated by a YXLON unit (Type TU 32‐D03, YXLON GmbH, Hamburg, Germany). For details of the dosimetric commissioning of this reference x‐ray irradiator please see Kuess et al.^26^ For the experiments described in this study, the following filtration was used: 3 mm Be + 3 mm Al + 0.5 mm Cu. The cell layer was positioned at 40 cm distance from the beam exit window (see Figure S1). Absolute dosimetry was conducted for this irradiation geometry with a Farmer type ionization chamber (T31013, PTW Freiburg, Germany) within a cell flask to account for attenuation of the plastic walls. Furthermore, dosimetric measurements were conducted using EBT3‐type Gafchromic films to verify dose homogeneity and with PinPoint Ionization chambers (T31015, PTW Freiburg, Germany) for absolute dosimetry, applying dose determination methodologies similar to patient‐specific quality assurance procedures.^26^, ^27^, ^28^

The size of the films was chosen to completely cover the slide of the chamber flask where the cells were growing. Using films in combination with the ionization chamber was beneficial to account for realistic x‐ray backscatter produced by the cell medium and the flasks itself. The dose homogeneity of the irradiated area of the chamber slide flask was within ±3%.

#### 2.B.2 Proton irradiation

The PMMA setup for proton irradiation was built to a depth of 40 cm to allow chamber slide flasks or detectors to be positioned along the entire range of clinically relevant energies up to 250 MeV. Multiple chamber slide flasks can be inserted and irradiated simultaneously. The remaining space of either unused slots or around inserted chamber slide flasks was filled with water to prevent range uncertainties caused by air gaps in the setup. A computed tomography (CT) scan of the setup in experimental condition (filled with flasks and water) was used for treatment planning. Using the TPS RayStation (V5.99, RaySearch Laboratories, Sweden), two different irradiation scenarios were planned, each with a SOBP of 80‐mm longitudinal dimension. The employed Monte Carlo code in the TPS RayStation 5.99 considers primary protons and secondary ions (protons, deuterons, and alpha particles). Primary and secondary protons are accounted for by class II transport method while the energy loss of heavier secondaries is approximated via a continuous slowing down approximation. In setup A, the more proximal target, the center of the SOBP was located at 80 mm, with beam energies ranging from 66.5 to 135.6 MeV to cover the entire SOBP. In the more distal located setup B, the center of the SOBP was positioned at a depth of about 155 mm with beam energies ranging from 127.2 to 185.9 MeV. The TPS has been specifically commissioned for the proton beam line in the research room at MedAustron following the procedures in the clinical beam lines.^29^ In contrast to the experimental validation of the dose calculation, LET_d_ calculations of the TPS have been validated against independent Monte Carlo particle transport simulations using GATE/Geant4, which itself also applies a dedicated proton beam model tracking all particles through the entire nozzle. The LET_d_ of the TPS agreed very well with the independent Monte Carlo simulations for all tested voxel sizes, where only a minor deviation (max. 5% at the distal edge) could be observed toward the end of the beam range due to the steep LET_d_ gradient and the moderate beam range differences of the TPS and the Monte Carlo simulations.^30^

Chamber slide flasks were positioned at a proximal, a central, and a distal SOBP position for each setup. More specifically, cell layers were located at 55, 80, and 105 mm in setup A, and at 130, 155, and 180 mm in setup B, respectively. Corresponding LET_d_ values were derived from Monte Carlo calculations performed with the TPS, based on the energy and beam spot information of the underlying treatment plan.^31^

### 2.C. Clonogenic survival assay

Standard clonogenic survival assays were performed after reference x‐ray or proton irradiation. Cells were harvested immediately after irradiation, diluted with supplemented medium appropriate for the cell line and seeded in 6‐well plate in concentrations according to the dose level: 250 cells (0, 0.5 Gy), 500 cells (1, 2 Gy), 1000 cells (4 Gy), and 2000 cells (6 Gy) per well, respectively. Following a cell line‐specific incubation period (7–14 days), cells were fixed with 96 % methanol, stained with 0.5% crystal violet solution, and colonies of more than 50 cells were considered as surviving clones.

### 2.D. RBE modeling and statistical analyses

Data points in all following tables and figures represent the mean values including the standard deviation of at least three independent experiments. Correlation of the parameters was tested using a F‐test on data fitted with linear regression. GraphPad Prism (GraphPad Software, Inc.) and Python 3.6 programming language (Python Software Foundation, https://www.python.org) were used for statistical procedures and the graphical illustration.^32^, ^33^

#### 2.D.1. Linear‐quadratic model

Based on the linear‐quadratic (LQ) formalism, surviving fractions in relation to the plating efficiency of nonirradiated control samples were calculated for each value of the delivered physical dose in Gy. The mean values and standard deviation results from a minimum of 18 individual values, corresponding to a minimum of three independent 6‐well plate per dose group. The margin of errors results from error propagation. A 1/σ‐weighted minimum chi‐square estimation was applied to the LQ model for survival curve fitting.^34^ Both parameters, α and β, were calculated for both radiation types using the same fitting method. For cells irradiated with protons, RBE values were calculated for the physical doses that reduced the cell survival to 90%, and 10%, respectively. To compare the experimental data to the model‐based predictions, RBEs were determined as a function of the LQ model parameters and the physical proton dose^35^:

(1) $$RBE\left(D_{p},\alpha_{x}\beta_{x},\alpha_{p},\beta_{p}\right)=\frac{\sqrt{\alpha_{x}^{2}+4\beta_{x}D_{p}\left(\alpha_{p}+\beta_{p}D_{p}\right)}-\alpha_{x}}{2\beta_{x}D_{p}}$$

*Calculation of the RBE*.^22^, ^35^

The Eq. (1) can be rewritten as a function of RBE_max_ and the RBE_min_ ^22^

(2) $$RBE\left(D_{p},{\left(\frac{\alpha }{\beta }\right)}_{x},RBE_{\max},RBE_{\min}\right)=\frac{1}{2D_{p}}\left(\sqrt{{\left(\frac{\alpha }{\beta }\right)}_{x}^{2}+4D_{p}}{\left(\frac{\alpha }{\beta }\right)}_{x}^{}RBE_{\max}+4D_{p}^{2}RBE_{\min}^{2}-{\left(\frac{\alpha }{\beta }\right)}_{x}\right)$$

*RBE as a function of the RBE_max_ and the RBE_min_*.

All applied phenomenological models have Eq. (2) in common but differ in their definition of the RBE_max_ and RBE_min_ functions. More details on the RBE models are summarized in the supplementary information (S3–S5). RBE values derived from the experiments and models were determined at dose levels of 0.5, 2, and 6 Gy.

The DSB model, with the endpoint of DSB induction (RBE_DSB_), predicts the repair kinetics of damaged cells considering the particle type, kinetic energy, and oxygen concentration. The computation was performed directly in the TPS (5.99) with Monte Carlo Damage Simulation software including the relation between particle LET and cell oxygen effects. The applied mechanistic RMF model, based on the auxiliary DSB model, can be well approximated by the LQ cell survival model for doses comparable or smaller than (α/ß)_x_ values. Parameters α and ß for proton radiation are then related to the reference radiation LQ parameters (α_x_, ß_x_) as well as to the outcome of DSB model RBE_DSB_ by Ref. [21]:

(3) $$\begin{array}{cc} \alpha_{p} & =\alpha_{x}RBE_{\mathrm{DSB}}\left(1+\frac{2{\bar{z}}_{F}RBE_{\mathrm{DSB}}}{{\left(\alpha /\beta \right)}_{x}}\right)\\ \beta_{p} & =\beta_{x}\;RBE_{\mathrm{DSB}}\;RBE_{\mathrm{DSB}} \end{array}$$

*LQ parameters for protons used in the RMF model*.

The term ${\bar{z}}_{F}$ describes the frequency mean‐specific energy that depends on the diameter of the cell nucleus and the nucleus mass density. Parameters given in Stewart et al.,^21^ were used for ${\bar{z}}_{F}$ calculations. The survival‐based RBE was subsequently computed using the Eq. (1).

## 3 RESULTS

### 3.A. Reference dosimetry and LET_d_

The dose homogeneity in both the 200 kV reference irradiation setup and the two proton setup was within 3% for the area covering a chamber slide flask. For the reference irradiation, the dose rate at the object surface was 1.28 ± 0.02 Gy/min. This value was measured with EBT3 radiochromic films, which were calibrated against a Farmer type ionization chamber in PMMA. Table I summarizes the LET_d_ values for proton irradiations, calculated directly in the TPS with Monte Carlo methods.

**Table I:** Sample positions within the irradiation setup with corresponding LET_d_ values for protons.

Relative position	Setup A energies: 66.5–135.6 MeV		Setup B energies: 127.2–185.9 MeV	
	Depth (mm)	LET_d_ (keV/µm)	Depth (mm)	LET_d_ (keV/µm)
Proximal	55	2.1	130	1.9
Central	80	2.8	155	2.5
Distal	105	4.5	180	4.1

**Fig. 1:**
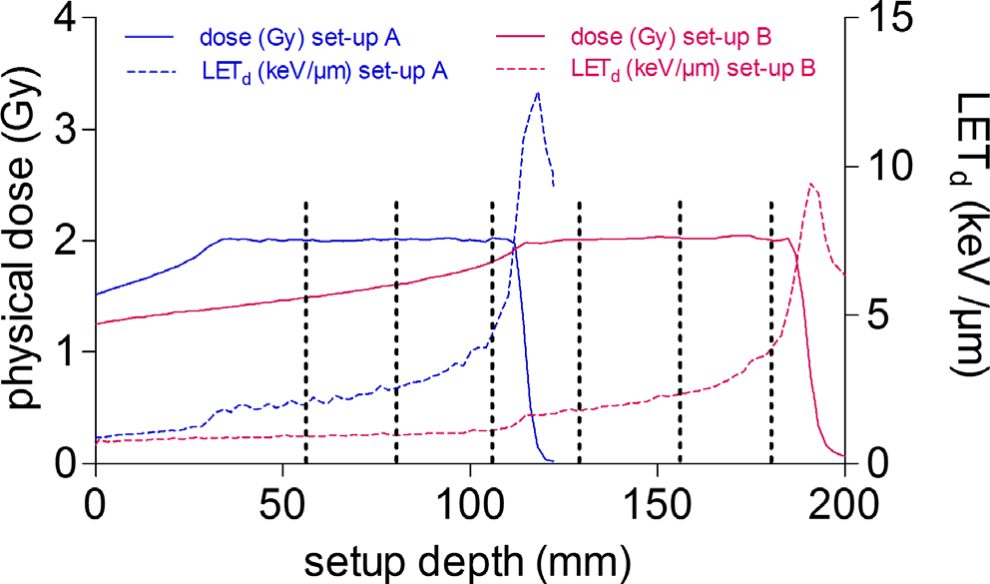
Central axis depth dose distribution of the proximal setup A (blue) and the distal setup B (pink), based on Monte Carlo calculation. The solid lines represent TPS data for both targets. The dashed lines illustrate the LET_d_. The SOBPs of both targets encompassed three positions each (proximal, central, and distal), indicated by black dotted stripes. [Color figure can be viewed at wileyonlinelibrary.com]

Figure 1 depicts the central axis depth dose distribution of setup A and setup B, including sample positioning and LET_d_, respectively.

### 3.B. X‐ray sensitivity

$\alpha_{x}$α_x_ values of 0.30 ± 0.02, 0.30 ± 0.02, 0.14 ± 0.02, and 0.12 ± 0.01 for HaCat, FaDu, Du145, and SKMel, respectively, were obtained from the x‐ray survival curves. Corresponding β_x_ values were 0.02 ± 0.00 (HaCat), 0.03 ± 0.00 (FaDu), 0.03 ± 0.00 (Du145), and 0.04 ± 0.00 (SKMel). The four cell lines were categorized according to their (α/β)_X_ ratios in high (α/β)_X_ cell lines (FaDu and HaCat, α/β ≥ 10) and low (α/β)_X_ cell lines (Du145 and SKMel, α/β < 5) for subsequent analysis in proton beams. The respective survival parameters are summarized in the left part of Table II.

**Table II:** LQ‐derived cell survival parameters and RBE values.

Cell line	200 kV x‐ray			Protons					
	α_x_ (Gy^−1^)	β_x_ (Gy^−2^)	α/β_x_ (Gy)	LET_d_ (keV/µm)	α (Gy^−1^)	β (Gy^−2^)	α/β (Gy)	expRBE_90_	expRBE_10_
HaCat	0.30 ± 0.02	0.02 ± 0.00	15.0	1.9	0.20 ± 0.03	0.04 ± 0.01	5.53 ± 1.73	0.72 ± 0.09	1.00 ± 0.02
				2.1	0.25 ± 0.09	0.03 ± 0.02	9.37 ± 9.30	0.84 ± 0.26	0.97 ± 0.05
				2.5	0.25 ± 0.06	0.03 ± 0.01	7.52 ± 4.66	0.87 ± 0.19	1.05 ± 0.04
				2.8	0.25 ± 0.11	0.04 ± 0.02	6.82 ± 6.73	0.87 ± 0.32	1.07 ± 0.05
				4.1	0.22 ± 0.03	0.04 ± 0.01	5.77 ± 1.78	0.79 ± 0.10	1.05 ± 0.02
				4.5	0.33 ± 0.06	0.02 ± 0.01	15.88 ± 10.37	1.13 ± 0.18	1.09 ± 0.03
FaDu	0.30 ± 0.02	0.03 ± 0.00	10.0	1.9	0.31 ± 0.02	0.03 ± 0.00	10.65 ± 2.51	1.04 ± 0.09	1.02 ± 0.02
				2.1	0.25 ± 0.02	0.03 ± 0.00	7.11 ± 1.19	0.85 ± 0.06	0.97 ± 0.02
				2.5	0.23 ± 0.01	0.04 ± 0.00	5.10 ± 0.41	0.80 ± 0.04	1.02 ± 0.01
				2.8	0.20 ± 0.03	0.05 ± 0.01	4.36 ± 1.33	0.72 ± 0.10	0.99 ± 0.02
				4.1	0.29 ± 0.04	0.04 ± 0.01	8.05 ± 3.01	0.99 ± 0.14	1.06 ± 0.03
				4.5	0.26 ± 0.01	0.04 ± 0.00	6.41 ± 0.56	0.90 ± 0.05	1.04 ± 0.01
Du145	0.14 ± 0.02	0.03 ± 0.00	4.7	1.9	0.34 ± 0.02	0.02 ± 0.00	18.51 ± 6.08	2.19 ± 0.22	1.28 ± 0.03
				2.1	0.30 ± 0.01	0.04 ± 0.00	8.34 ± 0.84	1.95 ± 0.17	1.37 ± 0.03
				2.5	0.31 ± 0.01	0.03 ± 0.00	10.25 ± 1.25	2.12 ± 0.19	1.39 ± 0.03
				2.8	0.33 ± 0.03	0.03 ± 0.00	10.22 ± 2.90	2.11 ± 0.24	1.39 ± 0.04
				4.1	0.37 ± 0.03	0.03 ± 0.01	10.97 ± 2.72	2.40 ± 0.26	1.51 ± 0.03
				4.5	0.39 ± 0.04	0.03 ± 0.01	12.54 ± 4.67	2.52 ± 0.29	1.53 ± 0.05
SKMel	0.12 ± 0.01	0.04 ± 0.00	3.0	1.9	0.32 ± 0.02	0.04 ± 0.01	8.67 ± 1.78	2.22 ± 0.21	1.36 ± 0.03
				2.1	0.35 ± 0.02	0.04 ± 0.00	9.15 ± 1.50	2.41 ± 0.22	1.43 ± 0.03
				2.5	0.29 ± 0.03	0.06 ± 0.01	4.71 ± 1.00	2.05 ± 0.23	1.49 ± 0.03
				2.8	0.43 ± 0.02	0.03 ± 0.00	12.84 ± 2.30	2.92 ± 0.25	1.56 ± 0.03
				4.1	0.34 ± 0.01	0.07 ± 0.00	5.20 ± 0.41	2.42 ± 0.19	1.64 ± 0.03
				4.5	0.42 ± 0.04	0.06 ± 0.01	6.60 ± 1.54	2.88 ± 0.23	1.76 ± 0.05

### 3.C. Proton sensitivity

**Fig. 2:**
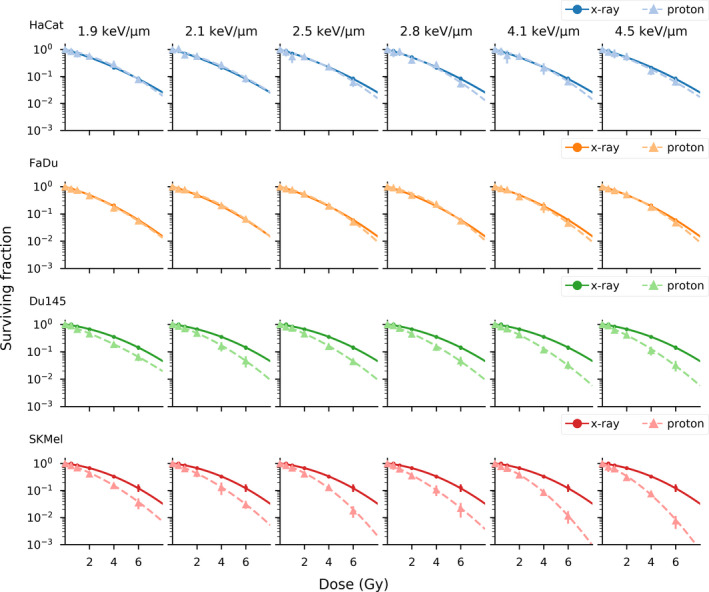
Cell survival curves of HaCat, FaDu, Du145, and SKMel after x‐ray (solid lines) and proton irradiation (dashed lines) sorted from the lowest to the highest LET_d_. Data points represent the mean values of a minimum of three independent experiments ± standard deviation. [Color figure can be viewed at wileyonlinelibrary.com]

After proton irradiation, survival curves and (α/β) parameters were calculated for all four cell lines, the respective survival parameters are summarized in Table II. Cell survival curves as a function of dose are depicted in Fig. 2, sorted from lowest LET_d_ to highest LET_d_ (also see Figure S6).

For HaCat and FaDu, the RBE values ranged from 0.72 ± 0.09 to 1.13 ± 0.18 for all levels of cell survival and for every LET_d_ value. In contrast, the low (α/β)_X_ cell lines, Du145 and SKMel exhibited RBE values substantially higher than the currently clinically used 1.1 for all levels of cell survival. For Du145 and SKMel cells, RBE values between 1.95 ± 0.17 and 2.92 ± 0.25 were obtained for the low dose range at 90% of cell survival and between 1.28 ± 0.03 and 1.76 ± 0.05 for 10% of cell survival (Table II).

**Fig. 3:**
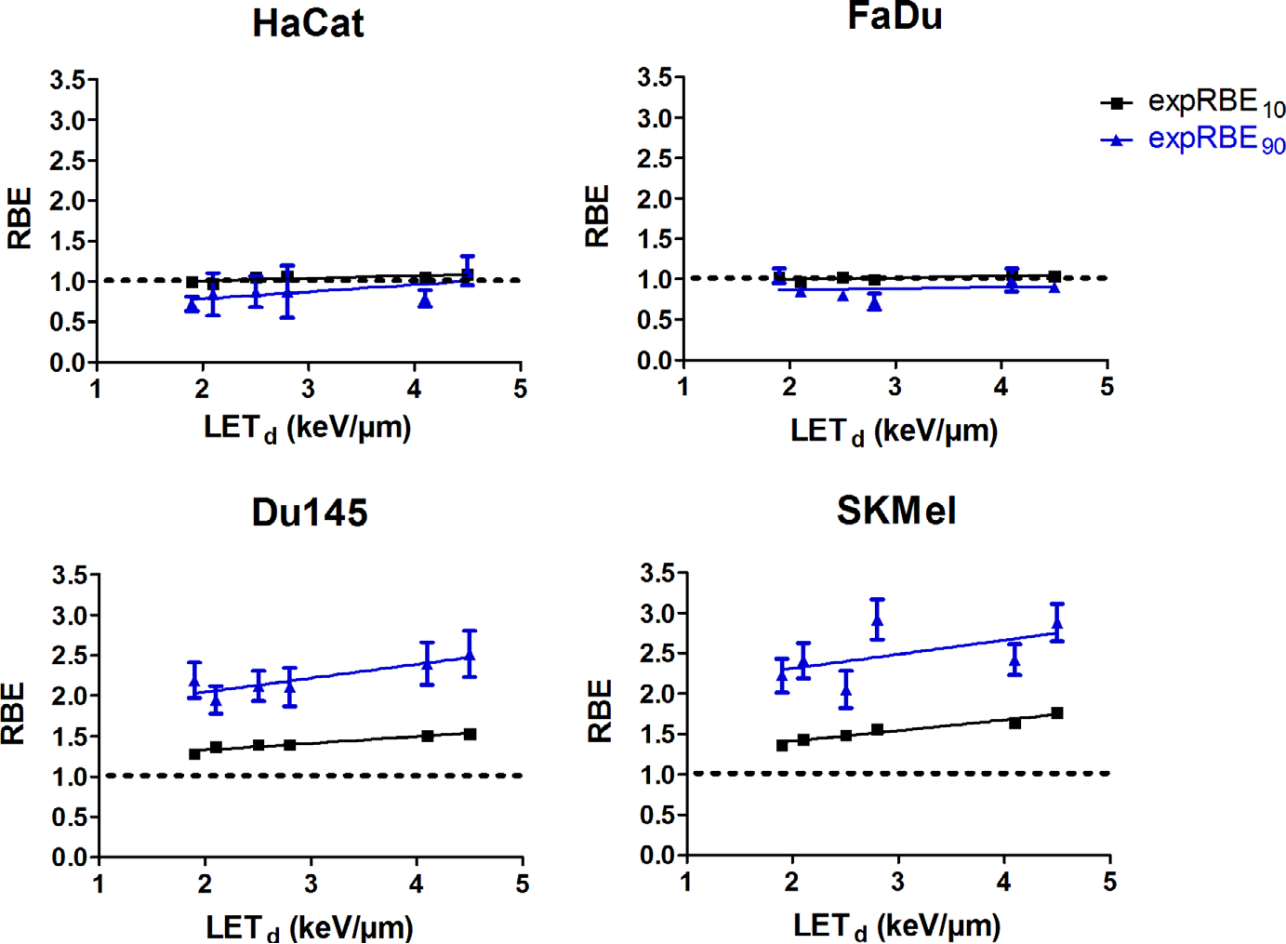
Relative biological effectiveness (RBE) values as a function of LET_d_ at cell survival levels of 10 % (black squares) and 90 % (blue triangles). The constant RBE level of 1.1 is illustrated with dotted lines. Linear fits were obtained from linear regression. Slopes were tested with *F*‐test. Data points represent a mean of a minimum of three independent experiments including the corresponding standard errors. [Color figure can be viewed at wileyonlinelibrary.com]

To test a potential correlation of LET_d_ and RBE, the experimental RBE values were fitted with linear regression (Fig. 3). No correlation was found for HaCat and FaDu. RBE values remained around unity across the whole investigated LET_d_ range. For HaCat, p‐values of 0.0834 (RBE_10_) and 0.1545 (RBE_90_) were calculated. F‐tests on FaDu linear regression fits resulted in p‐values of 0.1134 (RBE_10_) and 0.8265 (RBE_90_). A significant correlation between LET_d_ and RBE was found for Du145 at both levels of cell survival (RBE_10_ = *P* ≤ 0.0021, RBE_90_ = *P* ≤ 0.0183). SKMel displayed a significant correlation at RBE_10_ with *P* ≤ 0.0013 but not at RBE_90_ with *P* ≤ 0.2723.

**Fig. 4:**
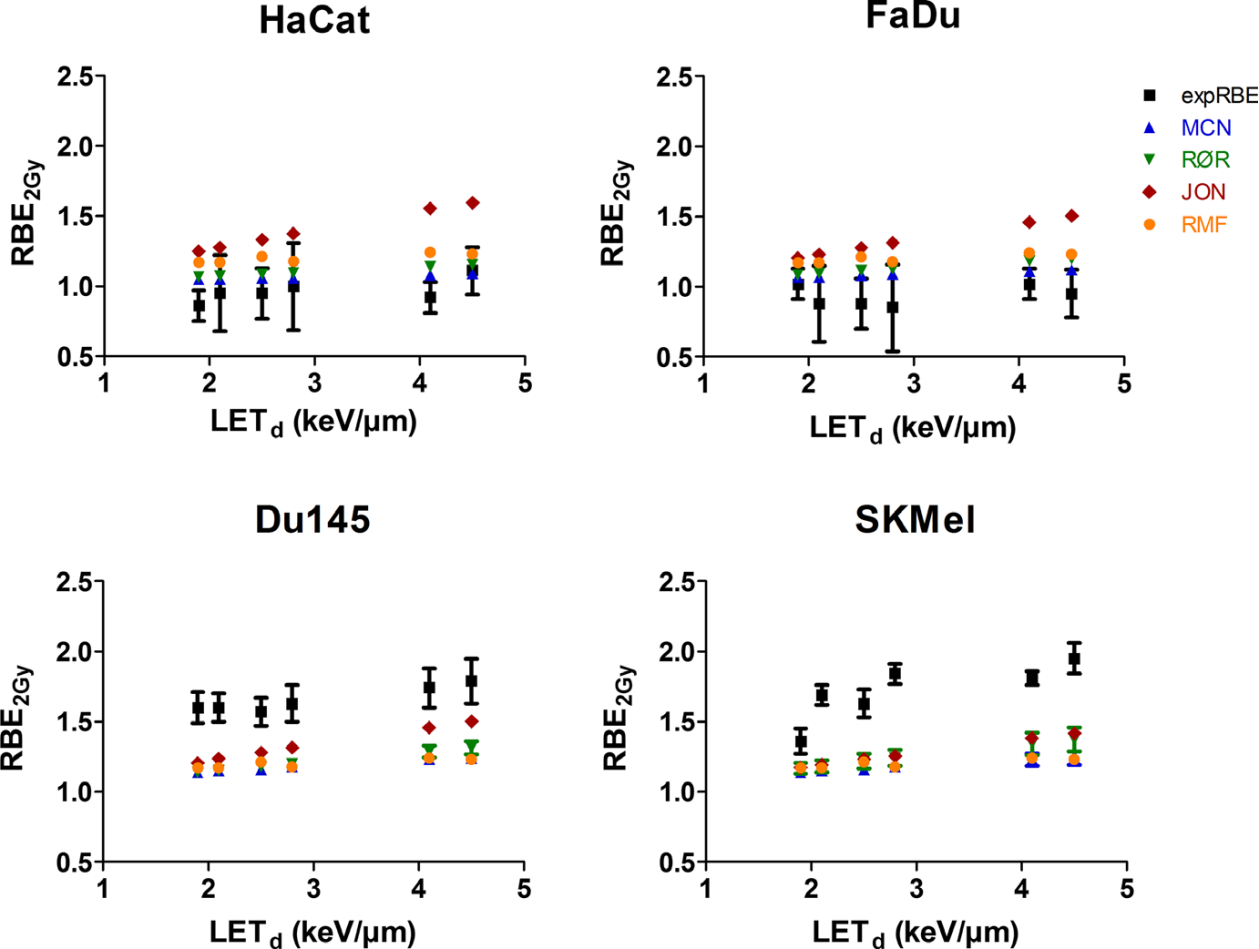
Experimental and model‐derived RBE_2Gy_ estimates as a function of the LET_d_. Data points represent a mean of a minimum of three independent experiments. The error bars indicate the estimated standard deviation obtained by error propagation of the measurement uncertainties on the alpha/beta values, for both, experimentally and model‐derived relative biological effectiveness values. [Color figure can be viewed at wileyonlinelibrary.com]

RBE model prediction at the dose level of 2 Gy is summarized in Table III and plotted against LET_d_ in comparison to the experimentally derived RBE_2Gy_ in Fig. 4.

**Table III:** Model‐derived RBE_2Gy_ predictions.

Cell line	LET_d_(keV/µm)	expRBE_2Gy_	MCN_2Gy_	RØR_2Gy_	JON_2Gy_	RMF_2Gy_
HaCat	1.9	0.84 ± 0.11	1.04 ± 0.00	1.06 ± 0.00	1.23 ± 0.01	1.16
	2.1	0.92 ± 0.27	1.05 ± 0.00	1.07 ± 0.00	1.25 ± 0.01	1.16
	2.5	0.92 ± 0.18	1.05 ± 0.00	1.09 ± 0.01	1.31 ± 0.01	1.20
	2.8	0.97 ± 0.31	1.06 ± 0.00	1.10 ± 0.01	1.35 ± 0.01	1.17
	4.1	0.89 ± 0.89	1.08 ± 0.01	1.14 ± 0.01	1.51 ± 0.02	1.24
	4.5	1.08 ± 0.17	1.08 ± 0.10	1.15 ± 0.01	1.56 ± 0.02	1.23
FaDu	1.9	1.02 ± 0.07	1.07 ± 0.00	1.09 ± 0.01	1.21 ± 0.01	1.16
	2.1	0.88 ± 0.07	1.07 ± 0.00	1.10 ± 0.01	1.23 ± 0.01	1.16
	2.5	0.88 ± 0.05	1.08 ± 0.00	1.11 ± 0.01	1.28 ± 0.01	1.20
	2.8	0.85 ± 0.10	1.08 ± 0.00	1.13 ± 0.01	1.31 ± 0.01	1.17
	4.1	1.02 ± 0.12	1.11 ± 0.01	1.18 ± 0.01	1.46 ± 0.02	1.24
	4.5	0.95 ± 0.05	1.12 ± 0.01	1.20 ± 0.01	1.50 ± 0.02	1.23
Du145	1.9	1.61 ± 0.11	1.11 ± 0.01	1.14 ± 0.02	1.21 ± 0.02	1.16
	2.1	1.61 ± 0.10	1.12 ± 0.01	1.15 ± 0.02	1.23 ± 0.02	1.17
	2.5	1.64 ± 0.13	1.13 ± 0.01	1.18 ± 0.02	1.28 ± 0.02	1.21
	2.8	1.64 ± 0.13	1.14 ± 0.01	1.20 ± 0.03	1.32 ± 0.02	1.18
	4.1	1.75 ± 0.14	1.19 ± 0.02	1.29 ± 0.04	1.46 ± 0.03	1.24
	4.5	1.81 ± 0.16	1.20 ± 0.02	1.31 ± 0.05	1.50 ± 0.03	1.22
SKMel	1.9	1.61 ± 0.09	1.14 ± 0.02	1.17 ± 0.04	1.17 ± 0.01	1.17
	2.1	1.69 ± 0.07	1.15 ± 0.02	1.18 ± 0.04	1.19 ± 0.01	1.17
	2.5	1.63 ± 0.10	1.17 ± 0.03	1.22 ± 0.05	1.23 ± 0.01	1.21
	2.8	1.84 ± 0.07	1.18 ± 0.03	1.24 ± 0.05	1.26 ± 0.01	1.18
	4.1	1.81 ± 0.05	1.23 ± 0.04	1.34 ± 0.08	1.38 ± 0.02	1.24
	4.5	1.95 ± 0.11	1.25 ± 0.05	1.37 ± 0.09	1.42 ± 0.02	1.23

All investigated models predicted, to varying extents, an increase of the RBE_2Gy_ with increasing LET_d_. RBE_2Gy_ calculations according to the MCN model resulted in values ranging from 1.04 to 1.12 for both high (α/β)_x_ cell lines (HaCat and FaDu) and from 1.11 to 1.25 for Du145 and SKMel. The RØR model predicted RBE_2Gy_ between 1.06 and 1.20 for HaCat and FaDu, and 1.14 and 1.37 for Du145 and SKMel. The JON modeling resulted in RBE_2Gy_ of 1.21 to 1.56 for HaCat and FaDu, and 1.17 to 1.50 for Du145 and SKMel. The mechanistic RMF model computed RBE_2Gy_ values between 1.16 and 1.24.

The MCN and RØR models assumed higher RBE_2Gy_ values for the low (α/β)_x_ cell lines Du145 and SKMel as compared to the high (α/β)_x_ cell lines HaCat and FaDu. The JON model predicted RBE_2Gy_ values of the same range for all cell lines and assumed the steepest increase of the RBE_2Gy_ with increasing LET_d_. The mechanistic RMF model, though being tissue‐specific, resulted in similar RBE_2Gy_ values for all four cell lines, irrespective of high vs low α/β_x_ values. MCN predictions for FaDu and HaCat agreed well with the experimentally derived RBE_2Gy_ values for most of the data points. Similar agreement, even though slightly worse than for the MCN model, was observed for the RØR and RMF models.

Off note, the model estimates were found to agree well within themselves for all RBE values within the investigated LET_d_ range. In contrast, all models underestimated the RBE_2Gy_ for the low (α/β)_x_ cell lines selected for our study, even though the model databases contain low (α/β)_x_ and low LET_d_ (< 5 keV/µm) data points.

## 4 DISCUSSION

With the increasing number of patients treated with PT, the concept of using a constant RBE of 1.1 is increasingly discussed but will probably remain an accepted approximation until the uncertainties concerning physical and biological parameters can be substantially reduced.^36^, ^37^ Recent publications raised concerns that a constant RBE is not beneficial to certain tissues and can cause adverse effects after PT.^12^, ^38^, ^39^

Most of the published studies on RBE dependencies focused only on one factor of influence and often assessed only one position within a SOBP. In addition, they were conducted using passive scattering, which historically was the most widely used beam delivering technique.^9^, ^40^, ^41^ This study aimed to overcome some of the limitations of previous investigations. The abovementioned considerable uncertainties in reported RBE from *in vitro* and *in vivo* studies can partly be attributed to nonstandardized experimental techniques and limited reporting on the experiment´s physical aspects, such as beam characteristics and dosimetry.

In order to reduce experimental uncertainties in radiobiological experiments for subsequent correlation between biological response and physical parameters, standardized irradiation setup for both, x‐ray reference and proton irradiation, were subjected to extensive dosimetric assessment prior to this study.^25^ The focus was set on clinically relevant proton energies and corresponding LET_d_ values. Finally, four human cell lines were selected to address differences in RBE dependencies for high and low (α/β)_X_.

An inverse relationship between RBE and dose per fraction was proposed^5^, ^11^, ^42^ and confirmed by our study, however, only for low (α/β)_x_ cell lines. We observed the highest RBE values in the low dose range for melanoma (SKMel) and prostate carcinoma (Du145) cells, both having an (α/β)_x_ < 5. The high (α/β)_x_ cell lines (HaCat and FaDu) did not show a similar response.

As far as LET_d_ dependencies of the RBE are concerned, multiple studies demonstrated a positive correlation and thus an increased RBE toward the distal end of a SOBP, even in proton beams.^43^, ^44^, ^45^ Our study confirms the LET_d_–RBE relationship, however, only for low (α/β)_X_ cell lines. In this context, our results complement previous studies, which suggest higher RBE values for tissues with low (α/β)_X_.^46^, ^47^ Wang et al., who assessed the impact of the human papilloma virus (HPV) status on the RBE of protons in different head‐and‐neck squamous cell carcinoma cell lines, report RBE_2Gy_ values between 1.15 and 1.19.^48^ Zlobinskaya et al. found a RBE_10_ of 1.1 in a FaDu xenograft model.^49^ Skin reactions following proton irradiation with moist desquamation as endpoint were quantified by Sørensen et al, who calculated RBE values between 0.9 and 1.06.^50^ RBE_2Gy_ values determined in our study are in agreement with this published data.

Less literature data are available for human low (α/β)_X_ lines and thus for benchmarking our melanoma or prostate carcinoma data. Petrovic et al. determined an RBE_2Gy_ in a range of 1.69 to 2.14 for their melanoma cell line, which agrees well with our date.^51^

The higher LET_d_ associated with scanned protons and range uncertainties might result in excess dose and LET_d_ outside of the PTV. In our study we tried to mimic clinical scenarios concerning the irradiation conditions, for example, the SOBP size and the target depth. Despite the low LET_d_ values resulting from clinically relevant proton energy ranges for an irradiation volume of 8 × 10 × 10 cm^3^ located at a depth of 80 and 155 mm, still substantial RBE variations could be observed. For tissues with high (α/β)_X_, the higher LET_d_ of scanned protons appears to be negligible. RBE values around unity, as found in this study and previously reported for tumors displaying high (α/β)_x_ values (e.g., medulloblastoma and head‐and‐neck malignancies), indicate a slight RBE overestimation in current practice.^46^, ^52^ Assuming that the cell/tissue sensitivity toward increased LET_d_ is determined by its (α/β)_X_ value, RBE values that differ considerably from the constant 1.1 can be expected toward the distal end of the SOBP, predominantly for low doses per fraction and low (α/β)_X_ ratio systems. Typical low (α/β)_X_ normal tissues adjacent to the PTV, such as the central nervous system, may need special attention in treatment planning given the current practice of treatment planning.

Based on the concerns regarding a constant RBE value of 1.1, several RBE models have been developed to predict RBE values taking into account the physical and biological factors. The phenomenological models are based on the widely accepted and used LQ formalism and incorporate tissue‐specific survival parameters, dose and LET_d_. Differences in input parameters and model assumptions, however, were recently shown to result in variations of the RBE predictions.^19^ In this study three phenomenological models were selected to predict the RBE values. The experimental *in vitro* data were systematically compared to model predictions. The applied phenomenological models assume an inverse relationship of RBE and photon‐derived (α/β)_X_. For the high (α/β)_x_ cell lines, all models slightly overestimated the RBE values. MCN predictions were the closest and mainly agreeing with experimental values within the statistical uncertainties. JON calculated the highest RBE, deviating the most from the experimentally derived RBE_2Gy_ values for FaDu and HaCat. The JON database consists of not only proton data but also experiments with heavier ions. The combination of particles in the modeling may result in higher RBE predictions for protons, as observed in our study. JON’s model is exclusively derived from experiments based on a small range of low (α/β)_x_ values and while deviating the most for the high (α/β)_x_ cell lines, the predictions deviate the least for the low (α/β)_x_ cell lines. When the phenomenological models were compared at different dose levels, it was noted that in‐between model agreement as well as prediction accuracy increased for higher doses (see results presented in supplementary material). Smaller errors as well as decreased deviation from the expRBE were obtained for survival at 6 Gy (RBE_6Gy_) as compared to 2 Gy (RBE_2Gy_) or 0.5 Gy (RBE_0.5Gy_), respectively (S7–S8).

The observed deviations of model‐derived RBE estimates and experimentally derived RBE values may be based on differences in model input parameters and/or reference irradiation. MCN and RØR accounted for the higher efficiency of low kV x rays and normalized their model input data to a 6 MeV radiation quality. In this study, we report RBE values derived from experiments with a 200 kV x‐ray reference beam. Rørvik et al. provide a systematic comparison of (α/β)_x_ values, LET_d_ inputs, and different RBE models. Two chosen phenomenological models MCN and RØR applied in this study were chosen based on their LET_d_ range which covers the low LET_d_ values (<5 keV/µm) relevant for our study. Furthermore, all chosen models include low (α/β)_x_ cell lines in their databases, but most of them are non‐human low (α/β)_x_ cell lines, such as the V‐79 Chinese hamster fibroblasts. Of all phenomenological models investigated, only the MCN model database contains one of our investigated cell lines, that is, the Du145 cell line. This fact may contribute to the differences between model predictions and experimentally derived RBE_2Gy_ values.

In addition to the three phenomenological models, one mechanistic model was applied. In order to evaluate a survival‐based endpoint with all models, the mechanistic model was fitted with the LQ model, based on DSB induction predictions (RBE_DSB_). RMF model RBE estimates aligned well with the phenomenological model predictions. Similar to all tested phenomenological models, the RMF model predicted the RBE well within uncertainties for the high (α/β)_X_ cell lines. No agreement of RMF estimates and experimentally derived RBE_2Gy_ could be observed for the cell lines with low (α/β)_X_, SKMel, and Du145.

This study focused on the irradiation response of 2D monocultures. This is in line with the fact that several biological optimization algorithms used in TPS for particle therapy, especially for heavier ions, are based on 2D *in vitro* data. Most of the studies concerning RBE determination are in essence done with 2D cultures because of their simple and fast handling and well‐established endpoint methods.^9^, ^44^, ^45^, ^46^ What differentiates this study from others is the application of clinically relevant doses and energy ranges and the use of a scanning pencil beam delivery system. However, multicellular 3D models of malignancies gain increasing importance in cancer research. Stromal disease components and 3D‐associated factors such as signaling gradients and diffusion as well as perfusion limitations contribute significantly to the therapy response *in vivo.* ^53^, ^54^, ^55^, ^56^, ^57^ These factors have not been considered in our study, as the main intention was to benchmark our data with already existing *in vitro* data and to specifically assess the RBE as a function of (α/β)_X_, derived from the basic LQ formula that is only applicable to 2D *in vitro* date.^58^

A major challenge remains if and how to account for the growing body of evidence that the RBE of protons is not constant. Accounting for the increase of LET_d_ with depth, similar to the clinical practice of carbon ion therapy, and the adaptation of an (α/β)_X_‐weighting factor could improve PT treatment planning and consequently safety and effectiveness. Given the reliance of treatment planning and modeling in both proton and carbon ion therapy on *in vitro* data, research to complement the increasing clinical utilization of particle therapy needs to build on high dosimetric accuracy and detailed reporting on relevant physical data. Only then model refinement can improve patient outcomes, but will directly depend on well‐characterized experiments to generate data with minimized physical and biological uncertainties.

## 5 CONCLUSIONS

The tissue‐specific fractionation sensitivity factor of photons, that is, the (α/ß)_X_ ratio, is a clear determinant of the RBE of protons and a predictor for its sensitivity toward LET_d_ changes. Higher RBE values than 1.1 can be expected for low (α/ß)_X_ tissues at proton beam end‐of‐range positions within the clinical practice of applied fractional doses. Current practice might slightly overestimate the RBE of protons for tissues with high (α/ß)_X_ ratios.

## FUNDING

Simon Deycmar is beneficiary of the ITN RADIATE funded by the European Union’s Horizon 2020 research and innovation program under the Marie Sklodowska‐Curie grant agreement (No. 642623) and supported by the Swiss State Secretariat for Education, Research and Innovation (SBFI, no. 15.0066).

## CONFLICT OF INTEREST

The authors have no conflict to disclose.

## Supporting information


**Fig. S1.** PMMA set‐up designed for reference X‐ray irradiation. Two culture flasks were irradiated simultaneously.
**Fig. S2.** Experimental set‐up designed for proton irradiation. Clinically relevant target energies were chosen, covering slots 3‐5 (set‐up A) and slots 6‐8 (set‐up B) in the PMMA block (a). CT images (b) were used for the treatment planning of the two SOPB positions. Accurate and standardized positioning was ensured with an in‐room laser system and a high precision robotic couch (c).
**Fig. S3.** Equations for the calculation of RBEmax and RBEmin used in the rewritten McNamara model.
**Fig. S4.** RBEmax and RBEmin equations used in the unweighted Rørvik model.
**Fig. S5.** RBEmax and RBEmin equations used in the Jones model.
**Fig. S6.** Cell survival curves of HaCat, FaDu, Du145, and SKMel after X‐ray and proton irradiation grouped per cell line. The lowest, a middle and the highest LETd investigated are graphically represented. Data points represent a mean of a minimum of three independent experiments ± SD
**Fig. S7.** Model‐derived RBE0.5Gy and RBE6Gy predictions in comparison to experimental results.
**Fig. S8.** Phenomenological model RBE prediction accuracy at different survival levels. Experimental RBE values (RBEexp) were compared to model‐derived RBE estimates at dose levels of 0.5 Gy (RBE0.5Gy), 2 Gy (RBE2Gy), and 6 Gy (RBE6Gy). Data points represent a mean of a minimum of 3 independent experiments ± standard errors.
