# Supplementary material for: Investigating the impact of alpha/beta and LETd on relative biological effectiveness in scanned proton beams: An in vitro study based on human cell lines
Source: Med Phys. 2020 May 15;47(8):3691–702. doi: 10.1002/mp.14212 (PMC7496287; doi:10.1002/mp.14212)
Supplement: Supplementary file 1 — Fig. S1. PMMA set‐up designed for reference X‐ray irradiation. Two culture flasks were irradiated simultaneously. Fig. S2. Experimental set‐up designed for proton irradiation. Clinically relevant target energies were chosen, covering slots 3‐5 (set‐up A) and slots 6‐8 (set‐up B) in the PMMA block (a). CT images (b) were used for the treatment planning of the two SOPB positions. Accurate and standardized positioning was ensured with an in‐room laser system and a high precision robotic couch (c). Fig. S3. Equations for the calculation of RBEmax and RBEmin used in the rewritten McNamara model. Fig. S4. RBEmax and RBEmin equations used in the unweighted Rørvik model. Fig. S5. RBEmax and RBEmin equations used in the Jones model. Fig. S6. Cell survival curves of HaCat, FaDu, Du145, and SKMel after X‐ray and proton irradiation grouped per cell line. The lowest, a middle and the highest LETd investigated are graphically represented. Data points represent a mean of a minimum of three independent experiments ± SD Fig. S7. Model‐derived RBE0.5Gy and RBE6Gy predictions in comparison to experimental results. Fig. S8. Phenomenological model RBE prediction accuracy at different survival levels. Experimental RBE values (RBEexp) were compared to model‐derived RBE estimates at dose levels of 0.5 Gy (RBE0.5Gy), 2 Gy (RBE2Gy), and 6 Gy (RBE6Gy). Data points represent a mean of a minimum of 3 independent experiments ± standard errors. [file MP-47-3691-s001.doc]

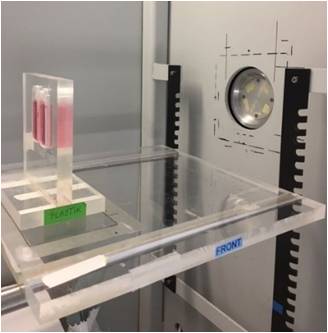


S1: PMMA set-up designed for reference X-ray irradiation. Two culture flasks were irradiated simultaneously.


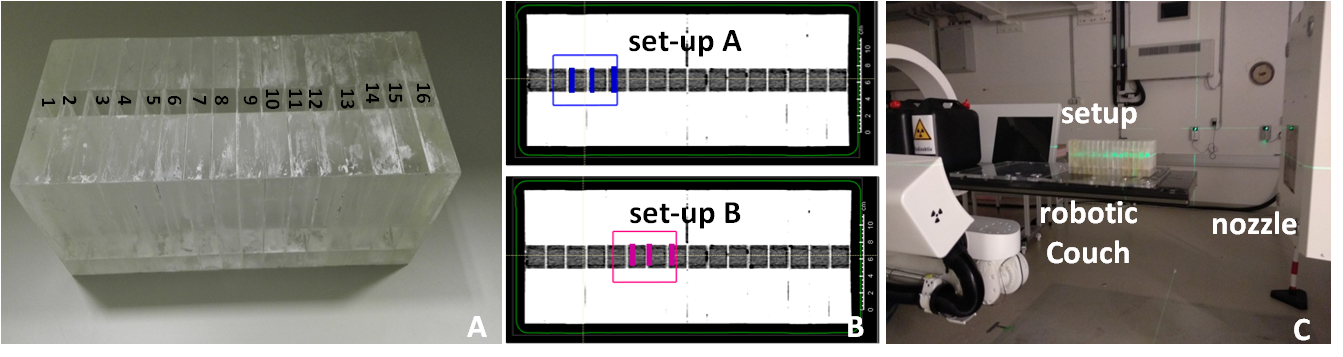


S2: Experimental set-up designed for proton irradiation. Clinically relevant target energies were chosen, covering slots 3-5 (set-up A) and slots 6-8 (set-up B) in the PMMA block (A). CT images (B) were used for the treatment planning of the two SOPB positions. Accurate and standardized positioning was ensured with an in-room laser system and a high precision robotic couch (C).


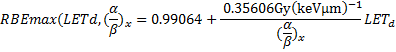


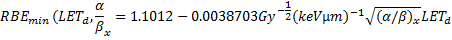


S3: Equations for the calculation of RBE_max_ and RBE_min_ used in the rewritten McNamara model.


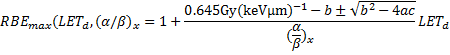


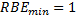


S4: RBE_max_ and RBE_min_ equations used in the unweighted Rørvik model.


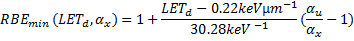


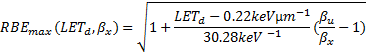


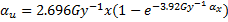


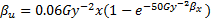


S5: RBE_max_ and RBE_min_ equations used in the Jones model.


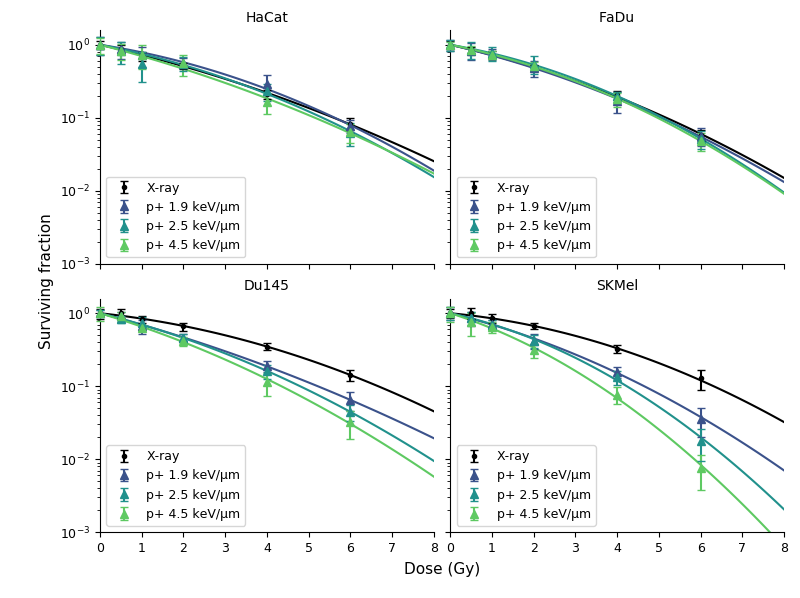


S6: Cell survival curves of HaCat, FaDu, Du145, and SKMel after X-ray and proton irradiation grouped per cell line. The lowest, a middle and the highest LET_d_ investigated are graphically represented. Data points represent a mean of a minimum of three independent experiments ± standard deviation.

S7: Model-derived RBE_0.5Gy_  and RBE_6Gy_ predictions in comparison to experimental results

| Cell line | expRBE_0.5Gy_ | MCN_0.5Gy_ | RØR_0.5Gy_ | JON_0.5Gy_ | expRBE_6Gy_ | MCN_6Gy_ | RØR_6Gy_ | JON_6Gy_ |
| --- | --- | --- | --- | --- | --- | --- | --- | --- |
| HaCat | 0.72 ± 0.11 | 1.04 ± 0.00 | 1.08 ± 0.01 | 1.27 ± 0.01 | 1.04 ± 0.13 | 1.05 ± 0.00 | 1.05 ± 0.00 | 1.17 ± 0.01 |
|  | 0.86 ± 0.26 | 1.04 ± 0.00 | 1.09 ± 0.01 | 1.31 ± 0.01 | 1.02 ± 0.28 | 1.05 ± 0.00 | 1.05 ± 0.00 | 1.19 ± 0.01 |
|  | 0.86 ± 0.20 | 1.05 ± 0.00 | 1.10 ± 0.01 | 1.37 ± 0.01 | 1.02 ± 0.16 | 1.06 ± 0.00 | 1.06 ± 0.00 | 1.22 ± 0.01 |
|  | 0.87 ± 0.35 | 1.06 ± 0.00 | 1.12 ± 0.01 | 1.42 ± 0.01 | 1.13 ± 0.29 | 1.06 ± 0.00 | 1.07 ± 0.00 | 1.25 ± 0.01 |
|  | 0.78 ± 0.11 | 1.08 ± 0.01 | 1.17 ± 0.01 | 1.63 ± 0.02 | 1.07 ± 0.13 | 1.07 ± 0.00 | 1.10 ± 0.01 | 1.37 ± 0.01 |
|  | 1.09 ± 0.20 | 1.09 ± 0.00 | 1.19 ± 0.01 | 1.69 ± 0.03 | 1.05 ± 0.16 | 1.06 ± 0.00 | 1.11 ± 0.01 | 1.41 ± 0.02 |
| FaDu | 1.03 ± 0.09 | 1.06 ± 0.00 | 1.11 ± 0.01 | 1.26 ± 0.01 | 1.02 ± 0.04 | 1.07 ± 0.00 | 1.05 ± 0.00 | 1.13 ± 0.01 |
|  | 0.85 ± 0.08 | 1.07 ± 0.00 | 1.12 ± 0.01 | 1.29 ± 0.01 | 0.92 ± 0.04 | 1.07 ± 0.00 | 1.06 ± 0.00 | 1.15 ± 0.01 |
|  | 0.80 ± 0.06 | 1.08 ± 0.01 | 1.15 ± 0.01 | 1.35 ± 0.01 | 0.98 ± 0.03 | 1.07 ± 0.00 | 1.07 ± 0.01 | 1.18 ± 0.01 |
|  | 0.72 ± 0.10 | 1.09 ± 0.01 | 1.16 ± 0.01 | 1.34 ± 0.01 | 1.03 ± 0.10 | 1.08 ± 0.00 | 1.08 ± 0.01 | 1.22 ± 0.01 |
|  | 0.98 ± 0.14 | 1.13 ± 0.01 | 1.24 ± 0.02 | 1.59 ± 0.02 | 1.07 ± 0.11 | 1.08 ± 0.00 | 1.12 ± 0.01 | 1.30 ± 0.01 |
|  | 0.89 ± 0.06 | 1.14 ± 0.01 | 1.26 ± 0.02 | 1.65 ± 0.02 | 1.03 ±0.03 | 1.09 ± 0.01 | 1.13 ± 0.01 | 1.33 ± 0.01 |
| Du145 | 2.05 ± 0.23 | 1.12 ± 0.02 | 1.21 ± 0.03 | 1.32 ± 0.02 | 1.26 ± 0.05 | 1.10 ± 0.00 | 1.07 ± 0.01 | 1.12 ± 0.01 |
|  | 1.90 ± 0.20 | 0.14 ± 0.02 | 1.23 ± 0.03 | 1.36 ± 0.02 | 1.39 ± 0.05 | 1.10 ± 0.01 | 1.08 ± 0.01 | 1.13 ± 0.01 |
|  | 1.92 ± 0.20 | 1.16 ± 0.02 | 1.28 ± 0.04 | 1.43 ± 0.02 | 1.31 ± 0.05 | 1.11 ± 0.01 | 1.09 ± 0.02 | 1.16 ± 0.01 |
|  | 2.02 ± 0.25 | 1.18 ± 0.02 | 1.31 ± 0.04 | 1.48 ± 0.02 | 1.34 ± 0.06 | 1.10 ± 0.01 | 1.10 ± 0.02 | 1.18 ± 0.01 |
|  | 2.22 ± 0.26 | 1.25 ± 0.03 | 1.45 ± 0.06 | 1.71 ± 0.03 | 1.40 ± 0.11 | 1.13 ± 0.01 | 1.15 ± 0.03 | 1.26 ± 0.02 |
|  | 2.32 ± 0.29 | 1.28 ± 0.03 | 1.49 ± 0.07 | 1.78 ± 0.04 | 1.43 ± 0.12 | 1.13 ± 0.01 | 1.16 ± 0.03 | 1.28 ± 0.03 |
| SKMel | 2.10 ± 0.14 | 1.18 ± 0.04 | 1.30 ± 0.07 | 1.31 ± 0.01 | 1.30 ± 0.09 | 1.11 ± 0.01 | 1.08 ± 0.02 | 1.09 ± 0.01 |
|  | 2.24 ± 0.14 | 1.20 ± 0.04 | 1.33 ± 0.07 | 1.33 ± 0.01 | 1.34 ± 0.03 | 1.11 ± 0.01 | 1.09 ± 0.02 | 1.10 ± 0.01 |
|  | 2.00 ± 0.18 | 1.23 ± 0.05 | 1.38 ± 0.08 | 1.40 ± 0.01 | 1.41 ± 0.09 | 1.12 ± 0.01 | 1.10 ± 0.03 | 1.12 ± 0.01 |
|  | 2.59 ± 0.15 | 1.26 ± 0.05 | 1.43 ± 0.09 | 1.45 ± 0.01 | 1.36 ± 0.03 | 1.13 ± 0.02 | 1.12 ± 0.03 | 1.13 ± 0.01 |
|  | 2.27 ± 0.12 | 1.34 ± 0.05 | 1.61 ± 0.13 | 1.66 ± 0.02 | 1.55 ± 0.02 | 1.14 ± 0.02 | 1.17 ± 0.04 | 1.19 ± 0.01 |
|  | 2.61 ± 0.21 | 1.39 ± 0.08 | 1.67 ± 0.15 | 1.73 ± 0.02 | 1.57 ± 0.08 | 1.15 ± 0.03 | 1.18 ± 0.05 | 1.21 ± 0.01 |

S8: Phenomenological model RBE prediction accuracy at different survival levels. Experimental RBE values (RBE_exp_) were compared to model-derived RBE estimates at dose levels of 0.5 Gy (RBE_0.5Gy_), 2 Gy (RBE_2Gy_), and 6 Gy (RBE_6Gy_). Data points represent a mean of a minimum of 3 independent experiments ± standard errors.
